# How to track cellular aging of mesenchymal stromal cells?

**DOI:** 10.18632/aging.100136

**Published:** 2010-04-08

**Authors:** Wolfgang Wagner, Simone Bork, Günther Lepperdinger, Sylvia Joussen, Nan Ma, Dirk Strunk, Carmen Koch

**Affiliations:** ^1^ Helmholtz Institute for Biomedical Engineering, Department of Cell Biology, RWTH Aachen University Medical School, 52074 Aachen, Germany; ^2^ Department of Medicine V, University of Heidelberg, 69120 Heidelberg, Germany; ^3^ Institute for Biomedical Aging Research, Rennweg 10, 6020 Innsbruck, Austria; ^4^ Reference and translational center for cardiac stem cell therapy, University of Rostock, 18057 Rostock, Germany; ^5^ Stem Cell Research Unit Graz, Medical University of Graz, 8036 Graz, Austria

**Keywords:** mesenchymal stem cells, replicative senescence, long-term culture, senescence marker, gene expression

## Abstract

Mesenchymal stromal cells (MSC) are currently tested in a large number of clinical trials and raise high hope in regenerative medicine. These cells have to be expanded in vitro before transplantation and several studies demonstrated that long-term culture evokes continuous changes in MSC: proliferation rate decays, the cell size increases, differentiation potential is affected, chromosomal instabilities may arise and molecular changes are acquired. Long-term culture of cell preparations might also have therapeutic consequences, although this has hardly been addressed in ongoing trials so far. Reliable therapeutic regimens necessitate quality control of cellular products. This research perspective summarizes available methods to track cellular aging of MSC. We have demonstrated that gene expression changes and epigenetic modifications are continuously acquired during replicative senescence. Molecular analysis of a suitable panel of genes might provide a robust tool to assess efficiency and safety of long-term expansion.

## Quality control for cellular therapeutics

There is growing interest in transplantation of *ex vivo* amplified cell preparations for various therapeutic applications. This has been fueled by novel insights from stem cell biology, new molecular tools and promising preclinical model systems. Mesenchymal stromal cells (MSC) can be isolated from various tissues including bone marrow and adipose tissue, which contain a rare population of adult stem cells (mesenchymal stem cells) with multilineage differentiation potential towards at least adipogenic, osteogenic and chondrogenic lineage [3]. To date, MSC are tested for a wide spectrum of diseases taking into account their paracrine effect, immunomodulatory activity and differentiation potential [4]. Hence, the use of MSC as cellular therapeutics necessitates stan-dardized isolation and reliable quality control of cell preparations. This, however, is greatly hampered by the multitude of different methods to prepare MSC [5]. Furthermore, there is a growing perception that even under highly standardized culture conditions, continuous effects during long-term culture and eventually replicative senescence need to be taken into account [2, 7].

**Figure 1.**
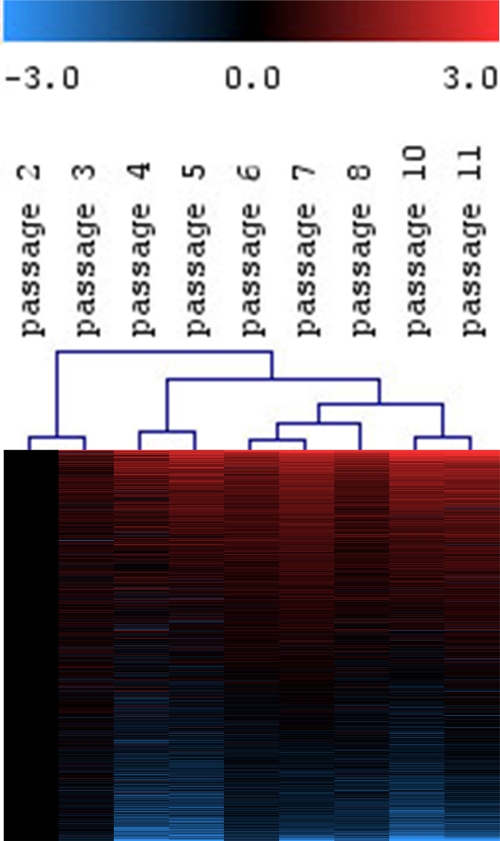
Continuous gene-expression changes in MSC upon long-term culture. MSC from human bone marrow were expanded for 11 passages and analyzed by Affymetrix GeneChip technology. Differential gene expression was always determined versus P2. Hierarchical cluster analysis of all expressed genes (19,448 ESTs) revealed continuous changes with higher passages. Hence, molecular changes in replicative senescence do not suddenly occur in late passages, but are acquired in the course of long?term culture.

MSC can only be culture expanded for a limited time before they reach a senescent state. This so called "Hayflick limit" is commonly observed in all primary cell isolates [8]. Senescent cells are mitotically arrested, thus are not dead, and remain metabolically active. However due to acquired functional and molecular changes, MSC increase in size, they adopt "fried egg morphology", expression of specific surface markers is attenuated [1] and adipogenic and osteogenic differentiation potential is affected [9-13]. Recently, we have demonstrated, that long-term culture is also associated with continuous changes in the global gene expression profile [1] (Figure 1). Genes involved in cell cycle, DNA replication, mitosis and DNA repair are significantly down-regulated in late passages. This reproducible pattern of senescence associated gene expression changes strengthens the hypothesis that cellular aging is driven by an organized process rather than a random accumulation of cellular defects [14]. Interestingly, long-term culture associated gene expression changes were related to age-associated changes in MSC from young *versus* elderly donors [15]. This indicates that cellular aging might be related to aging of the organism. The underlying molecular mechanisms of replicative senescence are still unraveled but it evidently has consequences for cellular therapy [2, 16]. However, it is not a trivial question how to track cellular aging of MSC.

## Restrictions of passage numbers and population doublings

Cells in culture can be continuously observed and hence, it appears straightforward to determine proliferation and the number of cell divisions. Obviously, the most convenient parameter for documentation of long-term culture is simply counting the number of cell passages. Under standardized culture conditions this procedure provides a predictive indicator for replicative senescence. However, as seeding densities often greatly vary between different laboratories (10 to 10^4^ cells/cm^2^) and also confluence at the time of harvesting [5, 2, 17, 18], the sole recording of passage numbers may lead to deceptive results in order to compare the state of senescence under non-standardized conditions. In this respect, calculation of the number of cumulative population doublings (PD) is more accurate [19]. MSC cultures are usually isolated by plastic adherent growth and hence, the initial MSC number can only be estimated by accounting fibroblastoid colony-forming unit (CFU-F) frequency based on the assumption that every colony has been derived from a single clonogenic MSC. Thereafter, cell numbers have to be exactly determined at all consecutive  passages as any inaccuracy will be carried over to the next passage and falsify PD. Yet, analysis of PD excludes the likely events of cells undergoing apoptosis, necrosis or loss during passaging. More importantly, there are big variations between different donor samples. Taken together, it is hard to predict at which passage or number of cell divisions MSC are approaching either a replicative or stress-induced senescent state.

## Surface molecules and histochemical markers for senescence

To date no specific molecular marker is available that prospectively reflects the degree of cellular aging in MSC. For instance the leptin receptor (CD295) increases with higher passages under hyperoxic culture conditions in MSC of elderly donors [20]. Flow cytometric analysis of this surface marker discriminates a CD295-positive subpopulation, but these cells also stained positive for annexin V. CD295 therefore stains apoptotic cells that accumulate at higher passages rather than senescent cells [20]. It is also possible to stain the enlarged senescent cells based on the accumulation of senescence-associated beta galactosidase (SA-β-gal). This lysosomal protein is predominantly active in senescent fibroblasts and also, albeit to a lower extent, in MSC [21]. The staining procedure is easy and reliable but the result can hardly be quantified and almost exclusively the very large senescent cells exhibiting a "fried egg morphology" stain positive for SA-β-gal [[1, 22]. It should be mentioned, that SA-β-gal itself is neither required nor causative for manifestation of senescence [23]. Despite limitations in quantification and prospective analysis of MSC, SA-β-gal is the most widely used biomarker for senescent and aging cells.

**Table 1.** Methods to track changes upon long-term culture.

**Method**	**Advantage**	**Disadvantage**
**Number of passages**	Counting of passages can be easily documented.	Seeding density and expansion techniques vary between different laboratories.
	Under standardized culture conditions it provides an indicator for long-term culture.	Even under standardized conditions there is variation between different probes.
**Cumulative population doublings**	PD can be calculated based on precise cell numbers at every passage and exact seeding densities.	The initial CFU-F frequency is required to estimate initial PD.
	This parameter is more robust for comparison between different laboratories.	MSC are heterogeneous and the number of PD does not correspond to the number of cell divisions in individual cells.
		Prospective information on the senescent state is hampered by large variation between different samples.
**SA-β-galactosidase **	Fast and easy method to stain activity of lysosomal, senescence associated beta-galactosidase.	SA-β-gal is not required for senescence.
	SA-β gal is over-expressed and accumulates specifically in senescent cells.	Especially the large cells become beta-gal positive.
		Quantitative analysis for quality control is difficult.
**Karyotype / array-CGH **	May detect mutations and potentially immortalized cell clones.	Human MSC appear to be relatively stable for karyotypic aberrations.
	Might prevent transplant-associated tumor formation.	No marker for normal cellular aging.
**Telomere length**	Might provide a direct measure for prospective analysis of potential cell divisions.	Stress induced senescence might be independent of cell cycle and telomere shortening.
	Several techniques are available to quantify telomere length.	It is yet unclear if analysis of telomere length facilitates reliable quality control in different MSC preparations.
**Gene expression markers**	RT-PCR and microarray techniques facilitate fast and reliable quantification.	Differential gene expression needs to be normalized to "house keeping genes".
	A panel of up-regulated and down-regulated genes may be more robust than individual markers.	Suitable gene-sets need to be established and cross-validated in different MSC preparations.

## Genomic aberrations

Clinical trials with MSC usually employ 1-2 x 10^6^ MSC per kg bodyweight for transplantation and therefore large-scale expansion is an indispensable prerequisite. Proliferation under non-physiologic in vitro culture conditions can result in mutations and chromosomal aberrations and eventually leads to malignant transformations. Karyotypic aberrations are commonly observed in MSC from mice and rats [24-26] whereas they have only been examined in few studies with human MSC [27-29]. So far tumor formation has not been described in clinical trials with MSC. Malignant transformation is obviously the "sword of Damocles" hovering above therapeutic cell products. The risk can be reduced by conventional karyotyping of MSC, however, minor genomic gains or losses may not be detected. Array complete genomic hybridization (CGH) analysisis more sensitive but this technique is incapable of revealing balanced translocations or very small mutations. Furthermore, malignant transformation may involve over-expression of c-myc, activation of cyclin dependent kinases, deletion of tumor suppressor genes such as p16^ink4a^, RB or p53 and re-expression of telomerase [30]. Initially, these changes may only occur in a small subset of cell preparations. At that point, it remains to be demonstrated whether routine karyotype analysis does actually reduce the risk of transplant-related tumor formation. It is however generally accepted that the stochastic effects of malignant transformation are no suitable markers in order to determine normal senescence-associated changes in MSC.

## Telomere length

MSC do not express telomerase and therefore telomere length decreases approximately 50-200 nucleotides per cell cycle [31] and there is evidence, that telomere shortening occurs also upon aging *in vivo* [33]. Ectopic expression of telomerase can immortalize MSC while their differentiation potential is maintained [32]. It is still under debate, if telomere loss really plays a causal role for replicative senescence or aging. Either way, loss of telomere length might facilitate some kind of internal clock to assess the state of cellular aging. Various methods are available to determine telomere length including Southern hybridization, flow cytometry based methods or quantitative PCR [34]. Therefore, telomere length may serve as another good indicator for mitotic history and the prospective additional life span. However, stress induced senescence may occur independent of cell division and it needs to be demonstrated if quantitative analysis of telomere length facilitates reliable and prospective quality control with regard to cellular aging.

**Figure 2.**
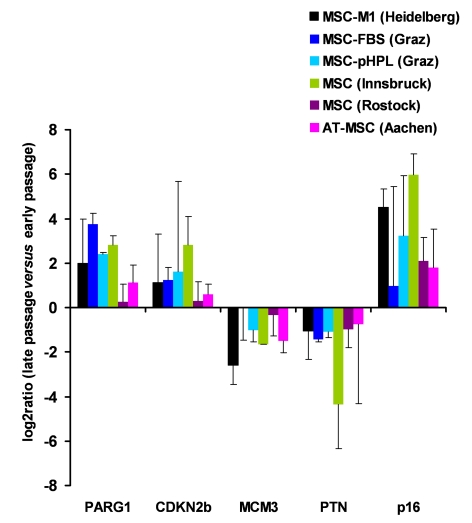
Gene expression markers for replicative senescence. MSC from human bone marrow were either culture expanded as described before in medium-M1 with 2% fetal calf serum (M1, in Heidelberg, Germany [1]; n=3), in culture medium with 10% fetal calf serum (FCS, n=2) or 10% pooled human platelet lysate (pHPL, n=2; both in Graz, Austria [38]), in MEM supplemented with 20% FCS (Innsbruck, Austria [40]; n=2), and in MSCGM (Lonza) culture medium (Rostock, Germany; n=4). Furthermore, MSC from adipose tissue were expanded with 10% pHPL (Aachen, Germany, n=4). RNA was isolated from corresponding early and late passages and analyzed for differential gene expression in PARG1, CDKN2B, MCM3, PTN and p16^ink4a^. Primers and methods have been described before [38]. These genes did not facilitate reliable discrimination of senescent cells in all samples but the tendency was consistent in all different MSC preparations.

## Senescence markers on gene expression level

Long-term culture induces continuous changes in gene expression [1]. A clear-cut characterization of distinct aberrations might facilitate determination when cells are shifting into the final state of senescence. A prerequisite is the reproducibility of senescence-associated gene expression changes in different MSC preparations, whereas techniques for cell isolation, culture media and cell culture methods have major impact on the composition of MSC and their gene expression profiles [35-37]. Recently, we have compared gene expression changes in MSC from human bone marrow, which had been isolated in two different laboratories, grown in long-term culture with different culture media and subsequently also analyzed with different microarray platforms [38]. Despite these differences there was a high resemblance in senescence-associated gene expression signatures. This led us to conclude, that these specific changes may be suitable for analysis of cellular aging. A matrix of distinctly up- and down-regulated genes thus provides a robust method for quality control. Taken together, we found senescence-associated up-regulation of the phosphate-associated RhoGAP protein-tyrosine (PARG1; alternatively termed ARHGAP29) and of the cyclin-dependent kinase-inhibotor 2B (CDKN2B). Genes that were down-regulated included pleiotrophin (PTN) and mini-chromosome maintenance complex component 3 (MCM3) (patent pending) [38]. Furthermore, work from other laboratories demonstrated that p16^ink4a^ is up-regulated at higher passages. We now performed quantitative RT-PCR analysis of these five genes in five different types of MSC preparations and long-term cultures from different laboratories (Figure 2). Overall, there were related changes when comparing early and senescent passage. However, standard deviations were rather high in these analyses and it was not always possible to discern MSC in late passage. As microarray technology facilitates simultaneous analysis of thousands of genes, a larger panel of genes most likely yields a more robust predictor for quality control purposes. Further specification of senescence-associated markers and cross-validation in different MSC preparations may pave the way for a reliable quality control of cell preparations on gene expression level.

Besides whole genome expression profiling, we could also demonstrate that DNA methylation profiles are clearly affected by long-term culture [2]. Using HumanMethylation27 BeadChip that represents 27,578 CpG sites in more than 13,500 annotated genes, it was shown that specific promoter regions become either hyper- or hypo-methylated upon expansion of MSC. Some of these deviations were also differentially methylated in fibroblasts (unpublished data). Diploid cells have only two copies of DNA, whereas gene expression is based on multiple copies of mRNA. Therefore, distinction of methylation changes is a potent way to monitor cellular aging and this type of epigenetic analysis could be more suitable for accounting the heterogeneity within primary MSC preparations and also with regard to cellular aging.

## Outlook

Cellular therapies are currently tested for various novel therapeutic applications. At the same time requirements for quality control of cell products have to be specified and standardized. Establishing efficient quality control is challenging as it bases on trial and error to accumulate knowledge on optimal culture conditions for therapeutic applications. So far there are only limited numbers of reports available tracking side effects of clinical application of MSC. Notably, some of the preliminary observations are very promising [4, 39]. It is yet unclear how many passages, population doublings or senescence-associated molecular changes are acceptable to grant optimal therapeutic effect for the different applications. Clearly, we need to establish a reliable method to track cellular aging of MSC. Molecular changes either on gene expression or DNA methylation levels provide powerful perspectives. Further bioinformatic analyses of datasets and validation enrolling different MSC preparations will pave the way for a reliable panel of distinct aging and senescence markers.
